# Who and how, DNA sensors in NETs-driven inflammation

**DOI:** 10.3389/fimmu.2023.1190177

**Published:** 2023-04-28

**Authors:** Félix-Antoine Aubé, Amel Bidias, Geneviève Pépin

**Affiliations:** ^1^ Département de Biologie Médicale, Université du Québec à Trois-Rivières, Trois-Rivières, QC, Canada; ^2^ Groupe de Recherche en Signalisation Cellulaire, Université du Québec à Trois-Rivières, Trois-Rivières, QC, Canada

**Keywords:** NETs, CGAS, TLR9, AIM2, NLRP3, inflammasome, sterile inflammation, NLRP3

## Abstract

During infections, neutrophil extracellular traps act like a meshwork of molecules that captures microbes. In contrast, during sterile inflammation the presence of NETs is usually associated with tissue damage and uncontrolled inflammation. In this context, DNA acts both as activator of NETs formation and immunogenic molecule fueling inflammation within the injured tissue microenvironment. Pattern recognition receptors that specifically bind to and get activated by DNA such as Toll-like receptor-9 (TLR9), cyclic GMP-AMP synthase (cGAS), Nod-like receptor protein 3 (NLRP3) and Absence in Melanoma-2 (AIM2) have been reported to play a role in NETs formation and detection. However, how these DNA sensors contribute to NETs-driven inflammation is not well understood. Whether these DNA sensors have unique roles or on the contrary they are mostly redundant is still elusive. In this review, we summarize the known contribution of the above DNA sensors to the formation and detection of NETs in the context of sterile inflammation. We also highlight scientific gaps needed to be addressed and propose future direction for therapeutic targets.

## Introduction

Neutrophils are polymorphonuclear leucocytes with a crucial role in the elimination of microbes during infectious inflammation. First described in 2004 (1), neutrophils extracellular traps (NETs), are a meshwork of inflammatory molecules, usually constituted by DNA, histones, neutrophil elastase (NE), high-mobility-group protein B1 (HMGB1), myeloperoxidase (MPO) and a plethora of primary and secondary granular proteins. NETs are produced by activated neutrophils, are usually degraded by DNases and are cleared by phagocytes such as macrophages. Overproduction of NETs or failure to clear them lead to their accumulation and subsequent tissue damage, organ malfunctions, uncontrolled inflammation and coagulation (2–4). Hence, the balance between NETs production and clearance is vital to homeostasis (5). First observed during infections (1), recent evidence shows that NETs are also produced during sterile inflammation, often leading to exacerbated inflammation and autoimmunity (6).

DNA is a critical component of NETs and is a potent immune stimulatory molecule. The clinical importance of DNA in inflammation is illustrated by autoinflammatory and autoimmunity symptoms observed in patients and in mouse models with deficiency in DNases (7–9). To stimulate immune responses, DNA binds to a class of pattern recognition receptors (PRR), called DNA sensors. Toll-like receptor 9 (TLR9), cyclic GMP-AMP synthase (cGAS), Absent in Melanoma 2 (AIM2) and NOD-like receptor protein 3 (NLRP3) (10, 11) were all shown to be activated by DNA in the context of NETs. Apart from cGAS, these DNA sensors are expressed in neutrophils and in the cell populations in contact with NETs. cGAS is absent from human and mouse neutrophils, but its downstream signaling adaptor protein Stimulator of Interferon Genes (STING) is present (12). Studies investigating the role of these DNA sensors rely on complete genetic deletion or on *in vitro* experiments using isolated cell populations, making it difficult to identify their direct and indirect roles in the formation and detection of NETs. This creates an important research gap and prevents us to decipher their specific involvement. Therefore, in this review, we discuss the current knowledge on the role of these DNA sensors in the formation of NETs and their detection during sterile inflammation.

## DNA sensors involved in the formation of NETs by neutrophils

TLRs are transmembrane proteins often located in endosomal compartments (endosomes, lysosomes or endolysosome) of myeloid cells such as macrophages and dendritic cells (DCs), playing a crucial role in host immune defense (13). Different types of nucleic acids activate different TLRs. While endosomal TLR7/8 (TLR13 in mice) are activated by RNA, TLR9 recognizes DNA with a preference for hypomethylated DNA which is primarily found in bacteria. Other proteins, like defensins and high mobility group box 1 (HMGB1) were shown to facilitate TLR9 activation by increasing DNA uptake (14–16). Canonical activation of TLRs leads to the production of inflammatory cytokines and of type-I Interferons (IFN-I), thus contributing to inflammation and immune responses (13). Inflammasome activation, which is strongly associated with the presence of NETs, relies on the activation of the NLRP family of cytosolic receptors. NLRP3 detects oxidized dsDNA (17, 18) and AIM2, a receptor from the interferon-inducible HIN-200 family, also detects dsDNA and can induce the formation of the inflammasome. Inflammasome activation results in the secretion of the interleukin IL-1β and promotes cell death. Neutrophils express both NLRP3 (19) and AIM2 (20) but only NLRP3 was shown to directly participate in the formation of NETs by neutrophils in the context of sterile inflammation, although the contribution of DNA in this activation was not investigated directly (19).

### TLR9 activates the formation of NETs by neutrophils

How TLR9 contributes to the formation of NETs by neutrophils is not fully understood. While TLR9 is usually located in the endosomal compartment, a study showed that primary blood neutrophils express a fully functional TLR9 at their cell surface that can detect extracellular DNA (21). *In vitro* experiments suggest that neutrophils are activated by CpG DNA and mitochondrial (mt)DNA but not nuclear DNA (22, 23). This mtDNA specific activation may be explained by the fact that mtDNA has a higher content of hypomethylated CpG DNA compared to animal nuclear DNA (24). Since high levels of circulating free mtDNA is strongly associated with inflammatory conditions (25), this could explain how neutrophils are rapidly being activated upon tissue injuries. Furthermore, activated neutrophils secrete interleukin-8 (IL-8), a chemotactic factor for neutrophils themselves, which then allow for the recruitment of more neutrophils in the inflamed tissue. As such, TLR9 activation could be an initiator event in NETs formation (26, 27). In support of this idea, a study reported that in a model of acute respiratory distress syndrome (ARDS), induced by the STING agonist diABZI, TLR9 contributes to neutrophils recruitment (28). STING activation results in cell death leading to cell-free (cf)DNA release, neutrophil recruitment and formation of NETs. DNase-I treatment or genetic ablation of TLR9 significantly reduces the recruitment of neutrophils (28). This suggests that cfDNA detection by TLR9 occurs prior to NETs formation. Again, mtDNA was the most abundant type of DNA detected in this context. Neither cGAS, AIM2 nor NLRP3 were involved in this response as genetic deletion of these genes did not significantly change neutrophils recruitment nor formation of NETs (28). A study suggested that activation of surface TLR9 on platelets could promote NETs formation by neutrophils. Activated platelets are important NET inducers in ANCA-associated vasculitis (AAV), a disease in which anti-neutrophil cytoplasmic antibodies are produced. *In vitro*, this is dependent on the secretion of CXCL4 (platelet factor 4-PF4) by platelets (29). The intertwined relationship between neutrophils and platelets has been brought to light in several reports (30, 31). Activated platelets stimulate the formation of NETs by neutrophils which in turn contribute to platelets activation, although the detailed molecular mechanisms and the immune receptors involved are still not fully defined.

The mtDNA-TLR9 axis in neutrophils activation and inflammation is well supported in the literature (32–34) and surface TLR9 activation of neutrophils by dsDNA has been linked to cases of cardiopulmonary bypass interventions (CPB). CBP leads to myoendothelial damages and increases levels of cfDNA including mtDNA in the plasma. This cfDNA binds to surface TLR9 of neutrophils to stimulate the formation of NETs (35). In a mouse model of primary graft dysfunction after lung transplantation surgery, TLR9 drives the formation of NETs following mtDNA release from cell death and tissue damages in bronchoalveolar fluid (36). *In vitro*, they show that mtDNA leads to a rapid release of citrullinated DNA complexes in a peptidylarginine deiminase 4 (PAD4) dependent mechanism. In addition to a role in NETs formation, results from graft experiments suggest that TLR9 has a broader contribution in NETs. Indeed, genetic ablation of TLR9 in the donor lungs or in the host mice significantly reduce the level of NETs (36).

## Receptors involved in the detection of DNA present in NETs

### TLR9 is activated by DNA containing-NETs

In addition to its role in direct neutrophils activation, TLR9 also contributes to the detection of NETs and therefore to NET-mediated inflammation. This is well illustrated in autoimmune syndromes with accumulation of immune-complexes containing nucleic acids like in Systemic Lupus Erythematosus (SLE). In pediatric SLE, NETs were found to contain DNA and LL37 peptides. This combination is taken up by plasmacytoid dendritic cells (pDCs) and lead to the production of IFN-I via TLR9 (37). In addition, the uptake of NETs by pDCs has been shown to stimulate the production of autoantibodies against NET components like DNA itself and HMGB1 (37). Interestingly the capacity of LL37 peptides to stimulate TLR signaling is not restricted to TLR9. In psoriatic skin, DNA and RNA in complex with LL37 peptide stimulate the production of NETs through TLR8/13 sensing in polymorphonuclear cells (37, 38). *In vitro*, neither RNA or nuclear DNA alone could mediate the production of NETs by polymorphonuclear cells, suggesting that following the formation of NETs, LL37 peptides are critical to NET sensing (38). In atherosclerosis, DNA from NETs contributes to the recruitment of more neutrophils through the TLR9 activation in macrophages and production of IL-8, confirming the pivotal role of IL-8 in this loop (39).

NETs also modulate wound healing and tissue repair through the activation of TLR9 in non-immune cells. In the context of diabetic foot ulcers, NETs prevent angiogenesis and delays wound healing. *In vitro* experiments have demonstrated that treatment of human embryonic vein endothelial cells (HUVEC) with NETs promotes endothelial-to-mesenchymal transition via activation of TLR9 (40). In a model of lung inflammation, chronic and excessive NETs formation contribute to pulmonary fibrosis through the activation of TLR9 in fibroblasts (41). Whether these TLR9 activation in non-immune cells are common feature of tissue damage remains to be determined.

### The emerging role of the cGAS-STING axis in NETs-driven inflammation

NETs are potent inducers of IFN-I specifically when the DNA is oxidized which renders it resistant to degradation and therefore more interferonogenic (42). As discussed above, TLR9 activation results in IFN-I production, but accumulating evidence confirmed that the DNA sensor cGAS is also a critical driver of IFN-I upon NETs detection (23, 43–48). Unlike TLR9, cGAS has not been detected in neutrophils, so its role is restrained to the detection of NETs rather than the formation of NETs by neutrophils (12). However, neutrophils express STING and the other effectors of the cGAS signaling pathway. Given that STING can be activated independently of cGAS, the effect of STING activation in neutrophils remains to be investigated.

The role of cGAS in driving IFN-I in response to DNA first came from a study trying to understand how mtDNA, which is abundant in the plasma of SLE patients, was driving IFN-I. In this study, they showed that injection of oxidized mtDNA in mice, induces a STING-dependent IFN-I response (43). However, in this setting, mtDNA was purified prior to the injection which could potentially mask the role of other NET components interacting with mtDNA. Likewise, in Neuromyelitis Optica Spectrum disorder (NMOSD), an autoimmune disease specific to the central nervous system, the IFN-I response correlates with the severity of the disease and with the presence of high levels of serum-derived cfDNA originating from NETs (48). The immunogenicity of this cfDNA was assessed *in vitro* using a combination of DNA sensor inhibitors and revealed that the IFN-I response was induced in a cGAS and TLR9 dependency (48).

A recent study has identified key mechanical insight into how cGAS detect DNA during NETs phagocytosis (23). Mechanistically, the authors revealed that after phagocytosis by human peripheral blood mononuclear cell (PBMC), DNA from NETs translocate to the cytosol where it activates cGAS. This is induced by the nuclear elastase, an enzyme responsible for the decondensation of chromatin. Using a model of autoimmune hepatitis induced by injection of the lectin concanavalin A, the authors demonstrate that NET induction resulted in cGAS-dependent stimulation of IFN-I response, suggesting that NETs also activate cGAS *in vivo* (23). The current view of the impact of DNase-I treatment is that it inhibits its immunogenicity. However, *in vitro* experiments showed that NETs treated with DNase-I display an increased capacity to activate cGAS compared to undigested NETs. This was observed until the DNA fragments reach the size of 150 bp, which is around the size of a nucleosome, when it stops to activate cGAS. Nucleosomes were indeed shown to inhibit cGAS activity (49). Unlike nucleosomes, other DNA binding proteins such as the mitochondrial transcription factor A (TFAM) or HMGB1 were shown to induce cGAS activation (50), suggesting that NETs components could modulate cGAS capacity to sense DNA. Given that several other studies have used DNase-I treatments without validating the type of DNA fragments they have produced, these results suggest that these data should be analyzed cautiously.

The contribution of cGAS-STING activation by cfDNA during tissue injury has been described in several models including in acute peripheral tissue trauma models, brain injury and strokes among others (44, 45, 51). Tissue plasminogen (tPA), used to breakdown blood clots in acute ischemic stroke, is associated with brain hemorrhage and the disruption of the blood brain barrier. In a recent study, it was revealed that treatment with tPA in a mouse model of stroke, significantly induces NETs markers such. DNase-I treatment or inhibition of NETs using PAD4 deficient mice significantly restores the integrity of the blood brain barrier. Genetic deletion of cGAS in mice also significantly reduces brain hemorrhage while on the contrary, cGAMP injection counteracts the effect of DNase-I treatment suggesting a critical role for the DNA sensor cGAS (45).

Finally, NETs have also been reported to play a role in the modulation of cancer cell behaviors (52, 53). For instance, in diabetic hepatocellular carcinoma (HCC), a cancer prone to metastasize, neutrophils invade the tumor and produce NETs, which then activate cGAS in HCC cells. Interestingly, HCC cells have a low level of expression of DNASE1L3, suggesting that NETs might not be degraded and eliminated properly (47).

### DNA-containing NETs activate the inflammasome

A contribution for AIM2 in NETS detection was reported in a lipopolysaccharide (LPS)-induced acute respiratory distress syndrome (ARDS) model during which intracellular NET-DNA binds the AIM2 receptor to activate the inflammasome ultimately resulting in alveolar macrophage pyroptosis (54). Another study reported the presence of AIM2 and the DNA binding protein IFI16 within NETs. It was shown that AIM2 binding to NETs protected these NETs from DNase-I degradation, suggesting a mechanism whereby extracellular AIM2-NET interactions may promote sustained IFN-I signaling (20). Although the authors did not show the origin of AIM2, it is possible that AIM2 is already present in neutrophils and released during NETs formation.

In psoriasis, NETs activate AIM2 inflammasome through the p38 MAPK signaling pathway, thus causing the production of IL-1β. However, treatment of NETs with DNase-I only slightly reduces the release of IL-1β, suggesting that this was independent of DNA. It was also found that NETs-activated AIM2 promoted the secretion of IFN-γ by keratinocytes, suggesting that DNA sensors behave differently in different cell populations (55). Furthermore, in the context of AIM2 in keratinocytes and psoriasis, LL37 peptides were shown to inhibit AIM2 dsDNA sensing (56). Interestingly, LL37 is also a transporter for cGAMP, the STING agonist produced by cGAS (57). In view of the involvement of cGAS in NETs detection, it would be interesting to determine whether the presence of cGAMP would interfere with the activation of inflammasome and whether it could promote STING activation in host targeted cells.

In macrophages, the activation of NLRP3-inflammasome was suggested to rely specifically on oxidized mtDNA (ox-mtDNA) while the AIM2-inflammasome could be activated by mtDNA and nuclear DNA (58). For instance, mtDNA accumulation in plasma of adult-onset Still’s disease activates NLRP3 in macrophages resulting in systemic inflammation and elevated level of IL-1β (17). Ox-mtDNA was shown to induce NLRP3 and the STING pathway in bone marrow derived macrophages. Genetic depletion of NLRP3 did not result in loss of the activation of STING by ox-mtDNA suggesting that STING activation is either independent or upstream of NLRP3 (59). Since prior studies showed that the cGAS-STING pathway can activate NLRP3, it would suggest that STING acts to upstream of NLRP3 in this context (60–62). Based on that, it would be interesting to define whether activation of NLRP3 by DNA is always STING-dependent.

## Discussion and future perspectives

**Figure 1:**
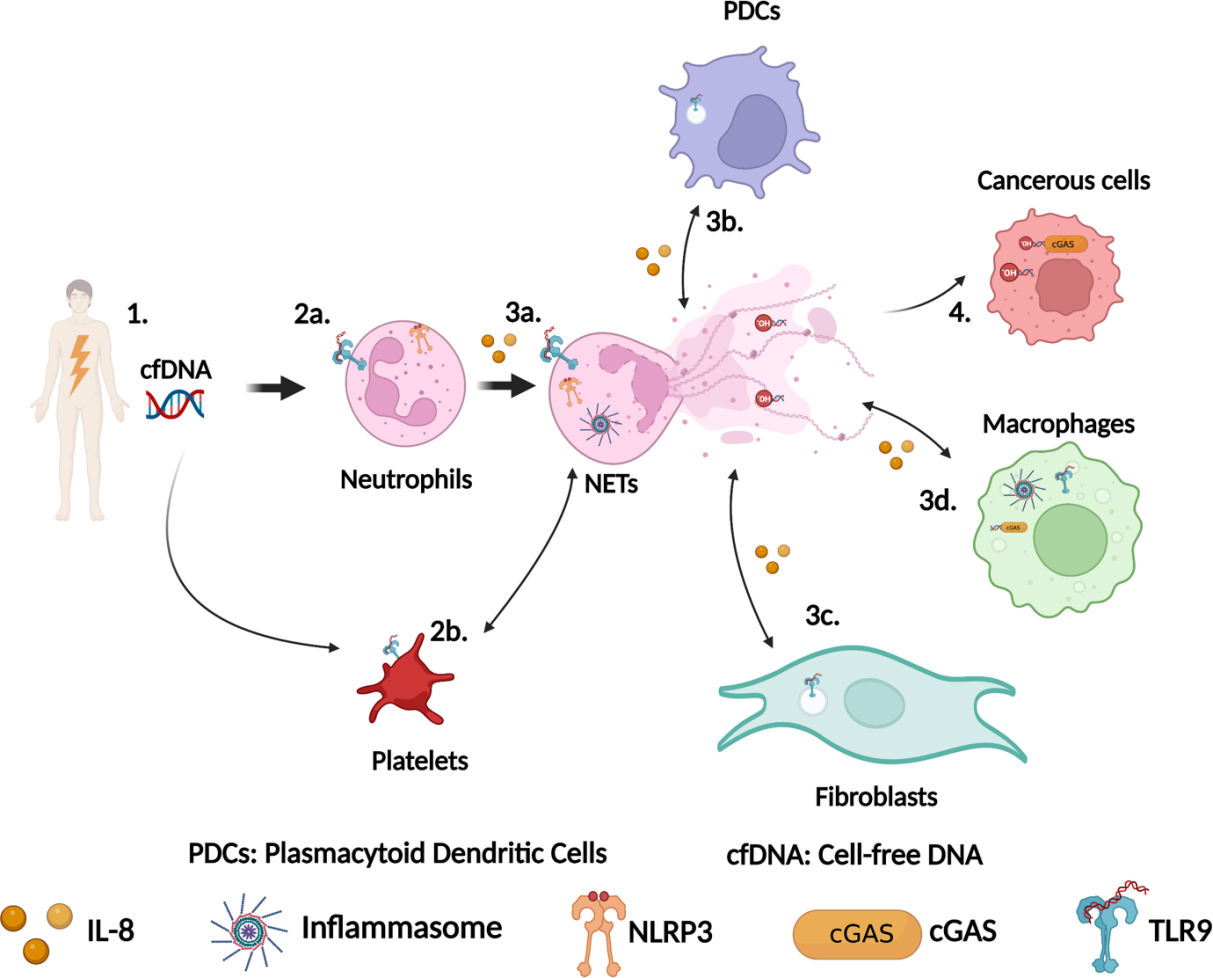
Schematic representation of the role of DNA sensors in NETs formation during sterile inflammation. (1) Tissue damage leads to cell-free DNA (cfDNA) release in the circulation. This cfDNA can originate from nuclei and mitochondria. (2a) Neutrophils detect cfDNA via their surface or intracellular TLR9 and/or via NLRP3. (2b) Platelets also detect cfDNA via surface TLR9 and activate neutrophils to produce NETs, which in turn drive platelets activation. (3a) Activation of TLR9 or NLRP3 leads to NETs formation. (3b) DNA from NETs is detected by TLR9 in plasmacytoid dendritic cells (PDCs) leading to cytokines production including IL-8, which trigger more neutrophils recruitment. (3c) Similarly, DNA from NETs can also be detected by TLR9 in fibroblasts driving their activation resulting in fibrosis. (3d) DNA from NETs can be phagocytosed by macrophages and sensed by cGAS or by the AIM2/NLRP3 inflammasome to stimulate the production of IFN-I response or the secretion of IL-1β. (4) Sensing of DNA from NETs by cGAS in cancerous cells modulate cancer cells behaviors. This figure has been created using BioRender.

The relevance of NETs in sterile inflammation is gradually emerging as illustrated in **Figure 1**. In pre-clinical models, accumulating evidence suggests that preventing NETs formation using inhibitor of PAD4 for example (45, 63) could benefit patients. Another strategy to improve patients’ health would be to directly target DNA sensors to prevent either NETs formation or detection. In order to do that, we need to identify precisely the involvement of each of these DNA sensors.

First, in which pathological context and tissue a given DNA sensor is activated. To date, most studies have selectively tested one or two DNA sensors, which prevent us from determining their specific contribution. In addition, the fact that TLR9 and cGAS activation result in a similar outcome in terms of cytokines produced like IFN-I suggests that they might have redundant and confounding effects. Many studies have reported the pathological roles of NETs in sterile inflammation without specifically investigating the role of these DNA sensors. For instance, a study established that the increased production of cfDNA in the airways characterizes a subset of neutrophilic asthma patients who have broad lung function impairments, poor symptom control with an exacerbation-susceptible phenotype (64). Although not characterized, the presence of cfDNA within the inflammatory airways is strongly suggestive to DNA sensors induced inflammation.

Second, DNA is not the only component of NETs. How the other NETs components such as LL37 influence the uptake of DNA and the activation of DNA sensors is still a core question that remains to be addressed. The composition of NETs varies according to the context and whether it is accompanied by neutrophil cell death in a process called NETosis needs to be defined (65). The DNA itself can be different depending on the presence of reactive oxygen species or of mtDNA (66–69). Although most studies that look into NET sensors have focused on DNA, recent studies showed that RNA derived from NETs is also immunogenic (38, 70).

Genetic depletion of TLR9 in neutrophils would allow us to determine whether TLR9 is involved in the first wave of neutrophils recruitment within the damaged tissue. Likewise, genetic depletion of STING in neutrophils will inform us on whether it has a cGAS independent role in the formation of NETs. Finally, given the controversial role of DNases in NETs immunogenicity, a more standardize use of DNases and the development of experiments to measure its activity *in vivo* may help understand the discrepancies in the outcome of DNases treatment. In summary, the fact that several DNA sensors contribute to NETs-driven inflammation offers a variety of targets for therapeutic interventions, however more studies are needed to clearly define the specific involvement of each DNA sensor.

## Author contributions

FA and AB wrote the first draft and edited the manuscript. FA designed the schematic. GP designed, wrote and edited the manuscript. All authors approved the final version of the manuscript.
